# Automated step detection with 6-minute walk test smartphone sensors signals for fall risk classification in lower limb amputees

**DOI:** 10.1371/journal.pdig.0000088

**Published:** 2022-08-18

**Authors:** Pascale Juneau, Edward D. Lemaire, Andrej Bavec, Helena Burger, Natalie Baddour

**Affiliations:** 1 Ottawa Hospital Research Institute, Ottawa, Canada; 2 Department of Mechanical Engineering, University of Ottawa, Ottawa, Canada; 3 Faculty of Medicine, University of Ottawa, Ottawa, Canada; 4 University Rehabilitation Institute, University of Ljubljana, Slovenia; 5 Faculty of Medicine, University of Ljubljana, Slovenia; Flinders University, AUSTRALIA

## Abstract

Predictive models for fall risk classification are valuable for early identification and intervention. However, lower limb amputees are often neglected in fall risk research despite having increased fall risk compared to age-matched able-bodied individuals. A random forest model was previously shown to be effective for fall risk classification of lower limb amputees, however manual labelling of foot strikes was required. In this paper, fall risk classification is evaluated using the random forest model, using a recently developed automated foot strike detection approach. 80 participants (27 fallers, 53 non-fallers) with lower limb amputations completed a six-minute walk test (6MWT) with a smartphone at the posterior pelvis. Smartphone signals were collected with The Ottawa Hospital Rehabilitation Centre (TOHRC) Walk Test app. Automated foot strike detection was completed using a novel Long Short-Term Memory (LSTM) approach. Step-based features were calculated using manually labelled or automated foot strikes. Manually labelled foot strikes correctly classified fall risk for 64 of 80 participants (accuracy 80%, sensitivity 55.6%, specificity 92.5%). Automated foot strikes correctly classified 58 of 80 participants (accuracy 72.5%, sensitivity 55.6%, specificity 81.1%). Both approaches had equivalent fall risk classification results, but automated foot strikes had 6 more false positives. This research demonstrates that automated foot strikes from a 6MWT can be used to calculate step-based features for fall risk classification in lower limb amputees. Automated foot strike detection and fall risk classification could be integrated into a smartphone app to provide clinical assessment immediately after a 6MWT.

## Author summary

Lower limb amputees have a high risk of falling. Despite this, most fall risk and prevention research focuses on healthy older adults. Artificial intelligence (AI) can be used to for fall risk analysis by identifying steps during walking for step-based feature calculation. However, the variability and instability of lower limb amputees make using traditional AI step detection methods ineffective, so manual labelling of steps was required. In this research, we validate the clinical application of a new deep learning step detection approach for lower limb amputees. Steps automatically-detected by a Long-Short Term Memory model were used to calculate features for fall risk analysis and compared to the previous method of using features calculated from manually-labelled steps. We demonstrate that both approaches correctly classify the same number of fall risk participants. Additionally, the approach using automated steps correctly classified over 80% of non-fall risk participants, though this approach did misclassify more participants who were not at risk of falls than the manual approach.

## 1. Introduction

Falls are the leading cause of death by unintentional injury in Canada [1] and the second highest cause worldwide [2], with adults aged 65 and older at the highest risk [3]. While most falls are non-fatal, injury and permanent disability are common. On average, 20% of falls experienced by older adults in the U.S. resulted in an injury [4]. Early identification and intervention for those at an elevated risk are critical to preventing falls and prolonging well-being. Screening tests, such as the Timed Up and Go (TUG), can be used to identify those at elevated risk of falling. While individual screening tests can be easy to administer and completed in minutes, they are often done in tandem with a battery of other movement assessments that can be time-consuming and draining for both patient and clinician.

Artificial intelligence (AI) has been proposed as a method for fall risk prediction and classification in the elderly by using wearable sensors to collect data during different movement assessments [5–7]. For example, waist-mounted triaxial accelerometer data collected during the TUG were used to train machine learning algorithms, such as logistic regression, to estimate postural stability and classify individuals as fall risk or non-fall risk [8]. Another commonly used movement assessment is the six-minute walk test (6MWT), which evaluates functional capacity and can be completed in most clinical settings. While the 6MWT is not clinically used for fall risk predictions, Drover et al. [9] used accelerometer data from the pelvis and shank during a 6MWT to train a random forest model to classify fall risk in an elderly population (73.4% accuracy). This demonstrated that richer knowledge can be extracted from a simple movement assessment instead of requiring multiple assessments from the patient.

While seniors account for a large proportion of fallers, people with lower limb amputations are also at elevated risk of falling at all stages of rehabilitation and also post-rehabilitation. People with lower limb amputations have highly variable gait patterns, even if they are very active [10]. This variability can result in greater instability, leading to a higher likelihood of falling than age-matched able-bodied populations [11]. Until recently, despite the high prevalence of falls, there was limited research available for classifying fall risk using artificial intelligence in the lower limb amputee population.

Daines et al. [12] proposed a method for fall risk classification in a lower limb amputee population using sensor data from a smartphone located at the posterior pelvis during a 6MWT. Most smartphones have integrated sensors (e.g., accelerometers, gyroscopes, magnetometers), are widely available, and are an accessible alternative to specialized dedicated equipment. Foot strikes from turns during the 6MWT were manually identified and used to calculate features from the smartphone signals to train a random forest model for fall risk classification (i.e., features calculated for each stride). The random forest model achieved 81.3% fall risk classification accuracy. However, manual foot strike labelling is time-consuming (i.e., project assistant inspected each acceleration signal and video data frame to identify each foot strike) and not viable for clinical use. Automated foot strike detection would improve the feasibility of implementing a fall risk classification model in a clinical setting. Rule-based algorithms can identify steps in elderly individuals with very high accuracy (99.95%) [13]. However, unstable and asymmetrical lower limb amputee gait makes foot strike detection challenging. There is limited research on heuristic models for foot strike detection in lower limb amputees, though the models are trained on data from a small number of participants, and often use sensors located at the lower limb [14,15]. Thibault et al. [16] explored a custom rule-based algorithm for foot strike identification in the same retrospective lower limb amputee 6MWT dataset as [12] using only the anterior-posterior (AP) linear acceleration signal. The rule-based approach resulted in 87% foot strike identification accuracy, noting that steps often needed to be removed, added, or relocated, suggesting that a more complex algorithm is required to analyze irregular gait patterns in lower limb amputees.

Recently, a novel method for automated foot strike detection in lower limb amputees using a deep learning approach was developed [17]. A long-short term memory (LSTM) deep learning approach was trained on the same retrospective lower limb amputee 6MWT dataset as [12]. Smartphone orientation, XYZ coordinates for raw and linear acceleration, and angular velocity collected from the posterior pelvis were used as input. The approach in [17] achieved 99.0% foot strike identification accuracy and stride parameters calculated from the automated foot strikes were equivalent to those of manually labelled foot strikes for most participants. These results demonstrated that automated foot strike methods can be used to calculate temporal features such as step time, stride time, and cadence for clinical decision-making. However, that study did not examine if the automated foot strike method is viable within a smartphone fall risk detection system.

In this paper, we determine if the automated step detection method in [17] can produce equivalent fall risk classification results with the random forest fall risk classifier in [12] that used manually labelled foot strikes, for people with lower limb amputations. If equivalent fall risk classification results are found with automated foot strike detection as with manually labelled foot strikes, this smartphone application could lead to enhanced 6MWT utility by identifying lower limb amputees who may be at risk of falling without using another specific fall risk test.

## 2. Methods

### 2.1 Participants

A convenience sample of 93 transtibial, transfemoral, and bilateral lower limb amputees were recruited from the University Rehabilitation Institute (Ljubljana, Slovenia) (Table 1). Clinical records provided self-reported number of falls, with falling at least once in the past six months prior to testing considered fall risk. The inclusion criteria were: transtibial or higher amputation; ability to walk with single cane, two crutches, or without any walking aids; minimum of six months post-amputation; had a functional prosthesis; no wounds on the residual limb; and was willing to participate. Participants who could not complete the full 6MWT test were excluded from analysis. Excluded trials were due to unknown fall risk status (8) and cell phone affixed to the side of the hip instead of lower back (5).

**Table 1:** Participant characteristics.

Age (years)	64.2 ± 12.2 (19–90)
Male	63 (78.8%)
Female	17 (21.2%)
Fall risk	27 (33.8%)
No fall risk	53 (66.2%)
Transtibial	72 (90.0%)
Transfemoral	3 (3.8%)
Bilateral (Transtibial)	5 (6.2%)
Time since amputation (years)	15.7 ± 18.0 (<1–65)
No aids	42 (52.5%)
Double crutches	25 (31.3%)
Single cane/crutch	12 (15.0%)
Rolling walker	1 (1.2%)

All participants provided written informed consent. This research was approved by the Ethic Committee of the University Rehabilitation Institute, Slovenia (# 46/2018) and re-approved for an additional 30 participants (# 27/2019). Each participant’s self-reported fall history was used for classifying participants as “no fall risk” or fall risk.

### 2.2 Data collection

**Fig 1:**
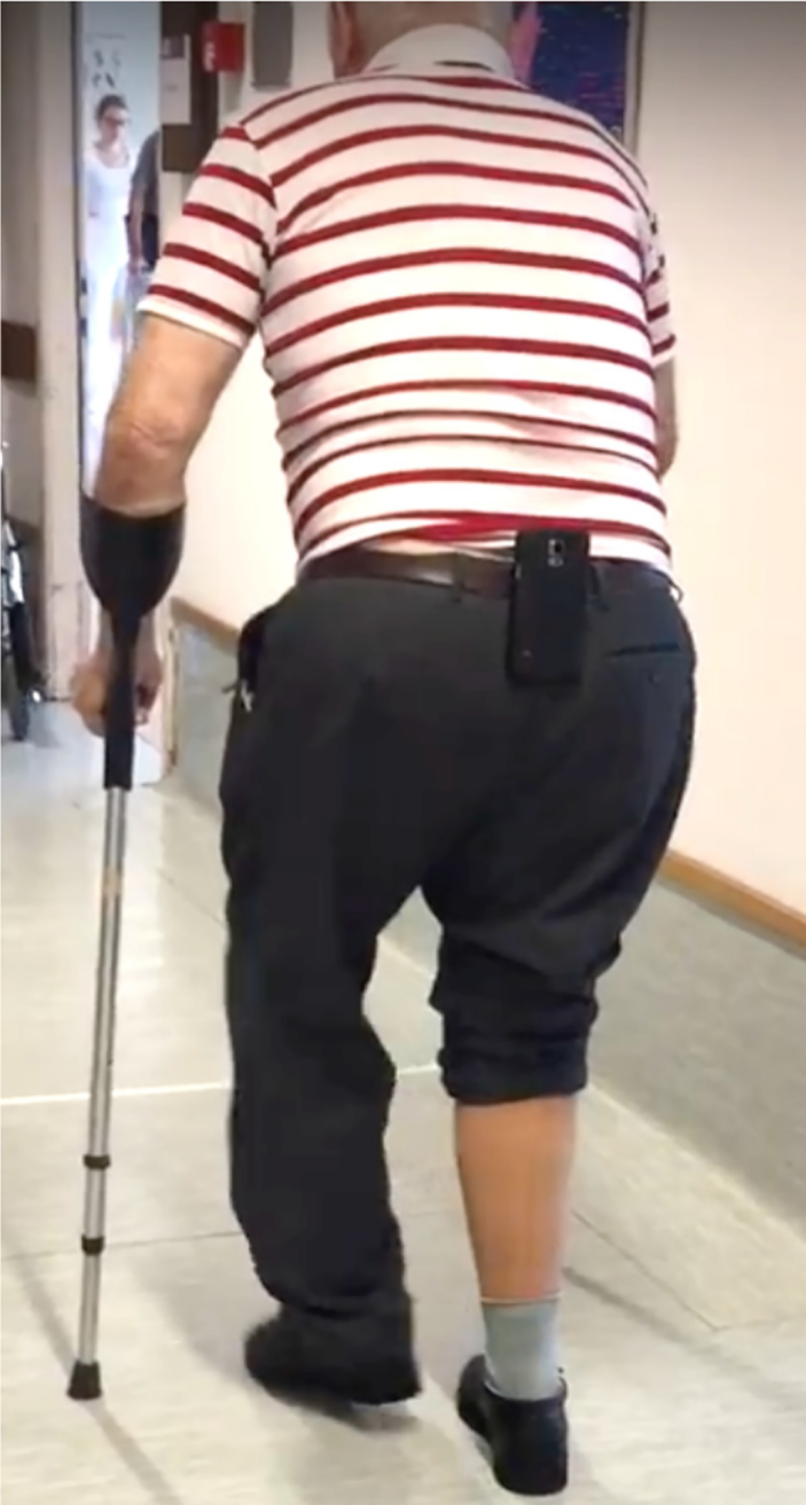
Experimental set-up.

An Android smartphone was placed on a belt at the lower back of each participant before completing a 6-minute walk test (6MWT) along a 20m hallway (Fig 1). Each participant completed one trial. Participants were video recorded during their assessment. Accelerometer, gyroscope, and smartphone orientation data were collected with The Ottawa Hospital Rehabilitation Centre (TOHRC) Walk Test app at 50 Hz [13]. Raw accelerometer data, gyroscope data, smartphone orientation, and timestamps for each recording were imported into MATLAB 2020b. Smartphone signals were re-interpolated to 50Hz using linear interpolation, then a fourth-order zero-lag Butterworth low pass filter with a cut-off frequency of 4 Hz was applied [18].

### 2.3 Step identification and fall risk classification

Ground truth steps were manually identified and labelled by two assistants prior to model training as label 0 (no foot strike present) and label 1 (foot strike present) using the following procedure. Smartphone linear acceleration over time was graphed. Foot strike events typically correspond to AP acceleration peaks followed by a vertical acceleration peak. Therefore, AP signal peaks immediately followed by a vertical signal peak were identified and the timestamp recorded as a foot strike event. Participant video was used to confirm foot strike identification. In cases where the foot strike event was not easily determined due to poor AP peak definition, double-peak, or irregular signal shape, the most appropriate location was determined by consensus. All other timestamps were consequently labelled as “no foot strike present”.

**Fig 2:**
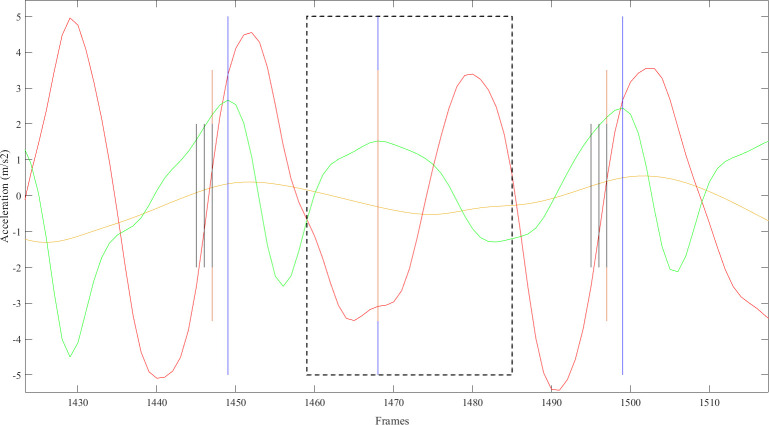
Example frame demonstrating a missed step identified within the locking period. A missed step (vertical blue line, frame 1468) identified within adjusted search range (black dotted line). A foot strike was inserted (vertical orange line, frame 1468) at timestamp corresponding to the peak AP acceleration (green curve) in this period.

The automated step-detection approach is described in detail in [17]. Smartphone 3D orientation, acceleration, and angular velocity signals from 6MWT trials were used as input data for an LSTM deep-learning approach for foot strike identification. Predicted foot strike labels were post-processed in MATLAB 2020b to correct for model prediction errors. This included extra foot strike predictions and missed steps. To identify missed steps, a method similar to that employed by Capela et al. [13] was applied. An adaptive locking period specific to each participant’s trial was defined from a 5 second sample of the filtered vertical acceleration signal from the beginning of the 6MWT trial. Periods where the duration between two consecutive steps was greater than 1.5 times the previous step were identified and searched for potential missed steps. The start of the period was increased by half the locking period, and end of the period was decreased by the same amount to prevent an inappropriately inserted step at the start or end of the original selected period. A foot strike was inserted at the timestamp for the peak AP acceleration within the adaptive locking period (Fig 2). Extra predictions were removed by identifying instances where two or more consecutive foot strike classifications occurred. The start and end of periods of consecutive predictions were located and the peak AP acceleration within the band was identified. The foot strike event corresponding to the AP peak was selected and all other predictions in this period were removed. Final cleaned predictions were used for feature calculation.

A random forest machine learning model developed by Daines et al. [12] was used for amputee fall risk classification. Random forests are machine learning algorithms that consist of multiple decision tree classifiers. Each decision tree in the ensemble provides an individual class prediction. The final classification is the class with the most predictions [19]. Features (i.e., variables calculated from the signal data) are used as input for each decision tree.

To evaluate the fall risk model using input based on manually labelled foot strikes or automated foot strike detection, two sets of features were calculated. Smartphone acceleration and angular velocity signals were used to calculate step-based features, using manually labelled foot strikes (M-FS) or automated foot strikes (A-FS). 62 features were extracted for each feature set (Table 2). Once features were extracted for each step, the minimum, maximum, mean, and standard deviation were calculated over all included steps for a total of 248 features (62 features multiplied by 4 statistics) per data set. These features were used in the random forest fall risk model to classify fall risk for each person, with results for A-FS and M-FS groups. Correlation-based feature selection (CFS) was used to reduce dimensionality, based on research completed in [12]. Leave-one-out cross validation was used to evaluate model performance.

**Table 2:** Feature list for fall risk classification. AP = anterior-posterior; ML = medio-lateral; RMS = root-mean square; FFT = fast Fourier transform; REOH = ratio of even/odd harmonic frequencies.

Temporal	Descriptive Statistics	Frequency Domain Features
Cadence	Minimum ML	Quartile FFT ML
Step time right	Minimum AP	Quartile FFT AP
Step time left	Minimum Vert	Quartile FFT Vert
Stride time	Maximum ML	Quartile FFT Tilt
Symmetry index	Maximum AP	Quartile FFT Rotation
	Maximum Vert	Quartile FFT Obliquity
	Mean ML	Maximum FFT ML
	Mean AP	Maximum FFT AP
	Mean Vert	Maximum FFT Vert
	Mean Tilt	Maximum FFT Tilt
	Mean Rotation	Maximum FFT Rotation
	Mean Obliquity	Maximum FFT Obliquity
	Range Tilt	Standard Deviation FFT ML
	Range Rotation	Standard Deviation FFT AP
	Range Obliquity	Standard Deviation FFT Vert
	Standard Deviation ML	Standard Deviation FFT Tilt
	Standard Deviation AP	Standard Deviation FFT Rotation
	Standard Deviation Vert	Standard Deviation FFT Obliquity
	Standard Deviation Tilt	Peak Distinction FFT ML
	Standard Deviation Rotation	Peak Distinction FFT AP
	Standard Deviation Obliquity	Peak Distinction FFT Vert
	RMS ML	Peak Distinction FFT Tilt
	RMS AP	Peak Distinction FFT Rotation
	RMS Vert	Peak Distinction FFT Obliquity
	RMS Tilt	REOH ML
	RMS Rotation	REOH AP
	RMS Obliquity	REOH Vert
		REOH Tilt
		REOH Rotation
		REOH Obliquity

Symmetry index: symmetry in right and left limb step times [20]

## 3. Results

For this study, 80 participants were suitable for fall risk classification, 27 fall risk and 53 no fall risk. Table 3 displays the fall risk classifier confusion matrices for A-FS and M-FS. The fall risk classifier trained on features calculated from M-FS correctly classified 64 of 80 participants and achieved 80% accuracy, 55.6% sensitivity, and 92.5% specificity. The A-FS approach resulted in 58 of 80 correctly classified participants and achieved 72.5% accuracy, 55.6% sensitivity, and 81.1% specificity. Classification of fall risk was the same for both approaches.

**Table 3:** Confusion matrices for automated and manual foot strike identification approaches.

Automated Foot Strike			Manual Foot Strike		
	No fall risk	Fall risk		No fall risk	Fall risk
No fall risk	43	10	No fall risk	49	4
Fall risk	12	15	Fall risk	12	15

Manual labelling was used as ground truth comparator for LSTM foot strike identification. For the participants included in this analysis, the LSTM foot strike identification model achieved 99.2% accuracy, 81.8% sensitivity, 99.7% specificity.

## 4. Discussion

This research demonstrated that automated foot strike identification is viable for smartphone-based fall risk classification with lower limb amputees. Fall risk classification was similar for both automated and manually labelled foot strike approaches, but the automated foot strike fall risk approach had more false positives (i.e., non-fall risk classified as fall risk). Automated foot strike detection is necessary in a clinical environment, where timely manual labelling is not feasible. A smartphone-based fall risk classification model from a 6MWT can benefit the patient and clinician since one assessment with a single sensor placement can provide functional capacity, stride parameters, and fall risk information to aid clinical decision-making.

The automated fall risk classification model could be used as a screening tool for lower limb amputees. Over 50% of fall risk participants and >80% of non-fall risk participants were correctly classified from signals collected during a 6MWT. The 6MWT is used to measure a person’s functional capacity, and fall risk information is not typically available from this assessment. A smartphone application integrating automated foot strike detection with fall risk classification could provide fall risk information to a clinician immediately after completing a 6MWT. However, to ensure that those who are at risk of future falls but were misclassified as no fall risk are not overlooked, patients could complete an additional assessment with higher fall risk sensitivity (e.g., TUG or L-test) to determine if an intervention strategy is necessary.

Six people who had not fallen in the 6 months before the study were misclassified as fall risk when features were calculated from automated foot strikes. A sub-group analysis of these participants did not identify any notable similarities that might explain their misclassification. They had varying ages, time since amputation, gait aid use, etc., mirroring the diversity of the people included in this study. Fall mechanisms are diverse and individual differences from person to person can contribute to the cause of a fall. More research is needed to better understand these mechanisms and determine what, if any, gait characteristics can be used to predict fall risk when using AI for fall risk classification.

Foot strike identification accuracy of the 6 participants misclassified as fall risk was also investigated. For these 6 participants, the LSTM foot strike identification model had 99.1% accuracy, 79.8% sensitivity, and 99.7% specificity. While the accuracy and specificity were similar to the classification results of the full dataset, sensitivity was 2% lower, representing a greater number of missed foot strikes. Errors in foot strike identification could have contributed to the misclassification of these participants. Increasing the training set size for foot strike identification and may help to improve foot strike detection and increase specificity for lower limb amputees.

The LSTM, with post-processing to correct errors such as extra predictions and missed steps, classified foot strike and non-foot strike events with 99.2% accuracy for the participants in this analysis. Other errors in foot strike identification included foot strike predictions that were within ±2 frames (±0.04 seconds). Some errors could not be corrected during post-processing, such as manually labelled steps that were not identified by the LSTM and extra foot strikes inserted in an inappropriate location. Despite these errors, clinical outcome measures, including stride parameters, were equivalent to manually labelled foot strikes for most participants.

A limitation of this research was the small number of people with transfemoral and bilateral amputations included in the dataset. While transtibial amputees are at a higher risk of falling in the post-operative period, people with transfemoral and bilateral amputations are at an elevated risk of falling post-rehabilitation [21,22]. A greater number of transfemoral and bilateral participants included in training and testing sets could improve fall risk model generalizability. Future research in this area would benefit from a greater number of transfemoral and bilateral amputees, with sub-group analysis.

## Conclusions

This study demonstrated that automatically detected foot strikes from a single smartphone sensor location on the body can be used to calculate step-based features for lower limb amputees after completing a 6MWT, leading to preliminary fall risk classification, an outcome that is not typically available for the 6MWT. This AI-enhanced 6MWT could be used to screen for people at risk of falls and then proceed with further assessments. Integration of this fall risk model into a smartphone application would improve the immediacy of the results, providing instant decision-making information. Future model development should include a greater number of people with transfemoral and bilateral transtibial amputations. This could improve the ability of the fall risk model to appropriately identify fall risk in these populations for further clinical assessment.
